# BMC3PM: bioinformatics multidrug combination protocol for personalized precision medicine and its application in cancer treatment

**DOI:** 10.1186/s12920-023-01745-y

**Published:** 2023-12-12

**Authors:** Majid Mokhtari, Samane Khoshbakht, Mohammad Esmaeil Akbari, Sayyed Sajjad Moravveji

**Affiliations:** 1 Department of Bioinformatics, Kish International Campus, University of Tehran, Kish Island, Iran; 2 Duke Molecular Physiology Institute, Duke University School of Medicine-Cardiology, Durham, NC 27701 USA; 3 Cancer Research Center, Shahid Beheshti University of Medical Sciences, Tehran, Iran

**Keywords:** Drug repurposing, Gene expression, Breast cancer, Microarray, Oncology

## Abstract

**Background:**

In recent years, drug screening has been one of the most significant challenges in the field of personalized medicine, particularly in cancer treatment. However, several new platforms have been introduced to address this issue, providing reliable solutions for personalized drug validation and safety testing. In this study, we developed a personalized drug combination protocol as the primary input to such platforms.

**Methods:**

To achieve this, we utilized data from whole-genome expression profiles of 6173 breast cancer patients, 312 healthy individuals, and 691 drugs. Our approach involved developing an individual pattern of perturbed gene expression (IPPGE) for each patient, which was used as the basis for drug selection. An algorithm was designed to extract personalized drug combinations by comparing the IPPGE and drug signatures. Additionally, we employed the concept of drug repurposing, searching for new benefits of existing drugs that may regulate the desired genes.

**Results:**

Our study revealed that drug combinations obtained from both specialized and non-specialized cancer medicines were more effective than those extracted from only specialized medicines. Furthermore, we observed that the individual pattern of perturbed gene expression (IPPGE) was unique to each patient, akin to a fingerprint.

**Conclusions:**

The personalized drug combination protocol developed in this study offers a methodological interface between drug repurposing and combination drug therapy in cancer treatment. This protocol enables personalized drug combinations to be extracted from hundreds of drugs and thousands of drug combinations, potentially offering more effective treatment options for cancer patients.

**Supplementary Information:**

The online version contains supplementary material available at 10.1186/s12920-023-01745-y.

## Introduction

Precision medicine and personalized medicine are emerging concepts in the field of healthcare that are revolutionizing the way diseases are diagnosed and treated [1]. These approaches can stratify patients and treat them according to their molecular characteristics [2]. Although precision medicine and personalized medicine are two different concepts, they complement each other in their application. Precision medicine is used to develop the accuracy of diagnoses and to refine patient classification [3]. It focuses on detecting the actual state of disease using laboratory tests to better understand pathological mechanisms. On the other hand, personalized medicine is a therapeutic approach that prepares or optimizes a specific therapy for an individual [4–6]. It uses computational frameworks such as drug repurposing, systems biology, and pharmacogenetics to discover appropriate individual treatments [7, 8]. The merging of these two approaches can lead to the era of personalized precision medicine [9, 10].

One of the key elements in precision and personalized medicine is understanding the unique gene expression patterns involved in diseases [11]. These patterns can be detected when studying patient populations, and they can be used to identify the most appropriate medicines that can regulate the genes involved in perturbed expression patterns in disease [12–14]. In particular, drug repurposing studies have shown great promise in accelerating the adoption of existing approved medicines that are in clinical use. This is because these medicines have already passed the rigorous safety tests required by regulatory agencies like the Food and Drug Administration [15–18].

Moreover, studies have shown that combination drug therapy can be more efficient in reducing drug resistance due to the control of parallel biological pathways. However, the heterogeneity of cancer leads to different responses to similar treatments. Therefore, it is essential to identify the gene expression pattern of each patient to determine the most effective combination drug therapy [19, 20].

To this end, we developed a bioinformatics protocol for personalized precision medicine by integrating the concepts of drug repurposing and drug combination. The protocol elucidates one or more drug combinations that are precisely adapted to individual gene expression profiles. This bioinformatics protocol helps the physician identify the most effective drug combination by precisely monitoring the patient's gene expression status.

**Fig. 1:**
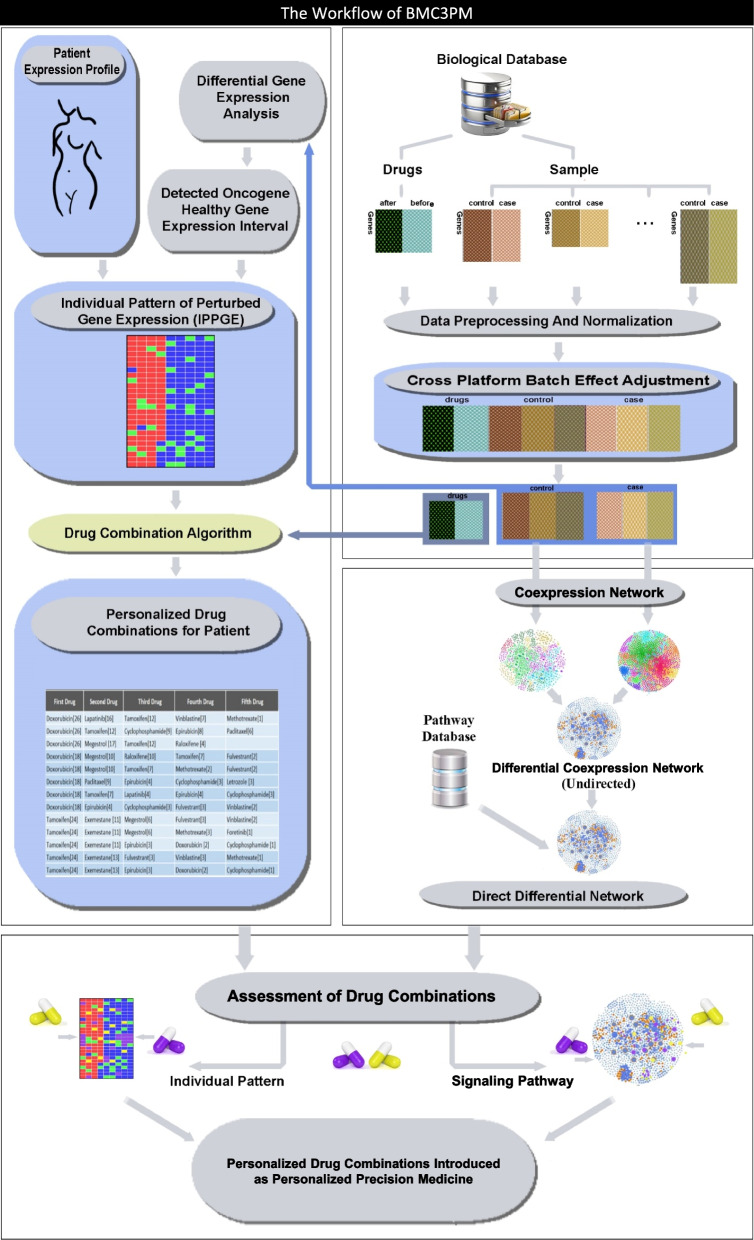
BMC3PM workflow. Bioinformatics Multidrug Combination Protocol for Personalized Precision Medicine (BMC3PM) is a set of various methods used in a bioinformatics protocol to extract personalized drug combinations

In this study, we aimed to apply our bioinformatics protocol to breast cancer patients. Firstly, we identified the differentially expressed genes (DEGs) in breast cancer patients compared to healthy controls. Secondly, we calculated a healthy gene expression interval for each DEG by assessing gene expression in healthy individuals. Thirdly, we compared a patient's gene expression profile with the gene health intervals (normal gene expression levels) to create an individual pattern of perturbed gene expression (IPPGE). Fourthly, we identified one or more drug combinations for the patient by simultaneously analyzing the IPPGE and the drug signatures through the use of a network-based algorithm. Finally, we reconstructed a directed differential network (DDN) using biological pathway data to predict the effect on gene expression of the identified drug combinations (Fig. 1).

In summary, the aim of this study was to demonstrate the feasibility and effectiveness of our bioinformatics protocol for personalized precision medicine. We believe that this protocol can be applied to other diseases and can contribute to the development of personalized precision medicine in the near future.

## Methods

The BMC3PM protocol was developed to extract a personalized combination of drugs that could effectively treat a patient's disease. BMC3PM is the integration of previously established methods along with the construction of new bioinformatics tools (Fig. 1).

### Data sets and Metadata information

The study analyzed gene expression data from 6173 untreated breast cancer patients and 312 healthy controls. The raw expression files (CEL files) for breast cancer patients and healthy controls were obtained using the GEOquery package in R, and all datasets were from the Affymetrix platform [21]. Supplementary Table S1 presents the sample size and platform information for each dataset. The study also used 691 drug perturbation gene expression profiles, which were downloaded from the Comparative Molecular Profiles of Approved Drugs (CMAP) databases and the National Center for Biotechnology Information (NCBI) databases (Supplementary Table S2).

### Pre-processing and normalization

Frozen robust multiarray analysis (fRMA) was used for background correction and normalization of the datasets. Unlike RMA normalization, fRMA is more suitable for clinical settings and can be applied to individual or small batch normalization [22]. The dataset was adjusted for downstream analyses by removing batch effects and unwanted variation among the GSEs using a combat function [23].

### Deregulated genes identification

**Fig. 2:**
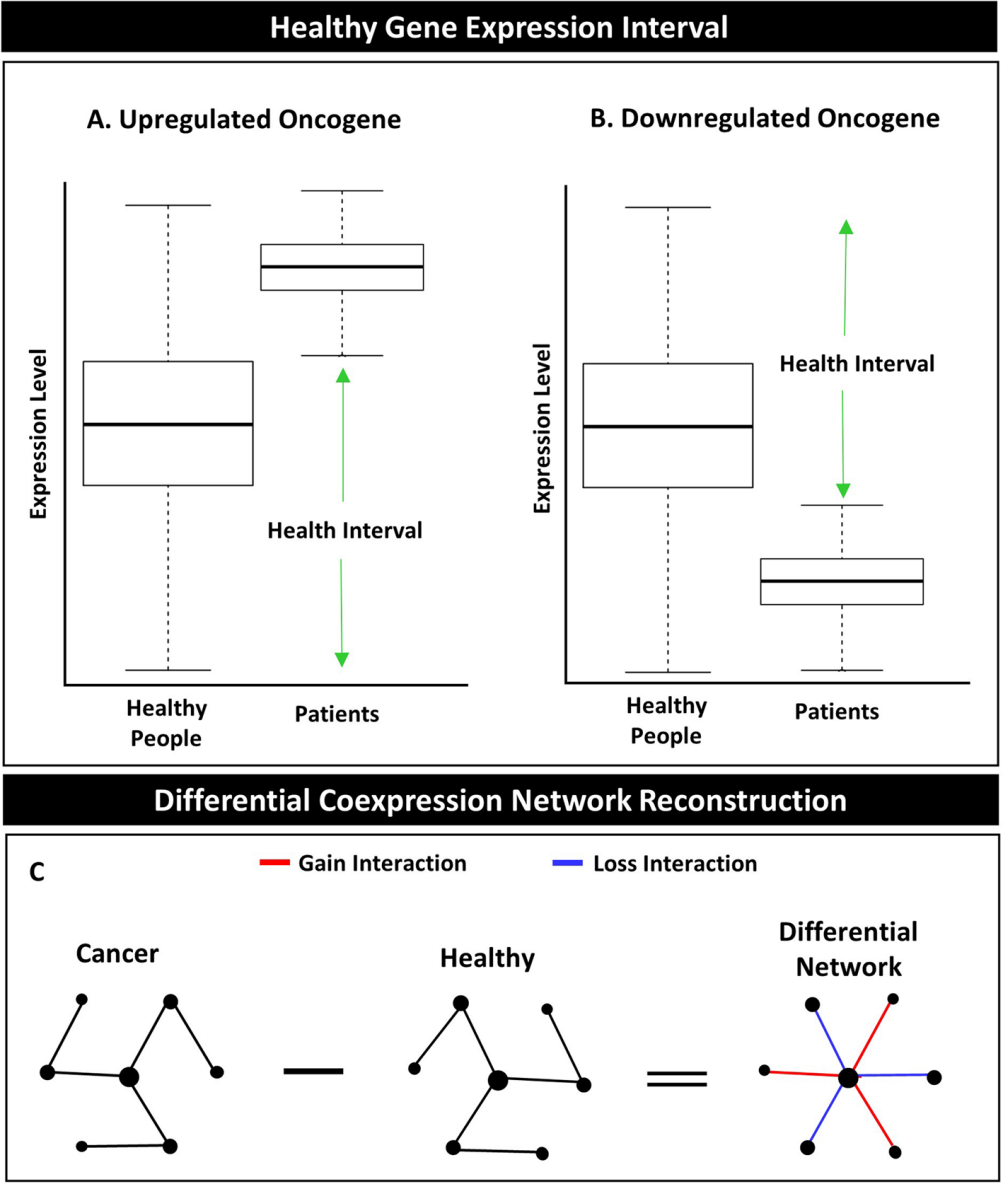
Data processing and analysis concepts. **A** The concept of the health interval for the upregulated genes. **B** The concept of the health interval for the downregulated genes. **C** The concept of the differential network is illustrated in cancer computational biology

After data pre-processing, differentially expressed genes (DEGs) were identified using the Limma package in R [24]. Deregulated genes were divided into two distinct groups: upregulated genes (URGs) and downregulated genes (DRGs) based on the LogFC. The LogFC of URGs was defined as greater than 0.45, and the LogFC of DRGs was defined as less than -0.45. The health intervals for each gene in the URG and DRG groups were calculated. The health interval refers to the range of gene expression in which members of the disease group are rarely seen within it [25] (Fig. 2 A and B).

### Upregulated Gene Health Interval (URGHI)

The quartiles of gene expression for the patient and control groups were calculated separately (without outliers) to identify the upregulated gene health intervals. Two main conditions were used to assess the quartiles of gene expression for the patient and control groups. Under the first condition, some genes' quartiles did not show overlap between the patient and control groups. Therefore, the range of gene expression between the minimum and maximum in the control group was selected as the URGHI. Under the second condition, some genes' quartiles showed overlap between the patient and control groups. Therefore, the range of gene expression between the minimum and third quartile in the control group was selected as the URGHI (Fig. 2A).

### Downregulated Gene Health Interval (DRGHI)

Two observed conditions were used to determine the downregulated gene health interval. Similar to the first condition for URGHI, the quartiles of some genes did not show overlap between the patient and control groups. Therefore, the range of gene expression between the minimum and maximum in the control group was selected as the DRGHI. In the second condition, the quartiles of some genes showed overlap between the patient and control groups. Therefore, the range of gene expression between the first quartile and maximum in the control group was selected as the DRGHI (Fig. 2B).

### Individual Pattern of Perturbed Gene Expression (IPPGE)

Each patient's disease-leading genes (individual deregulated genes) were identified using the health intervals calculated in the previous step and the individual's gene expression profile. Accordingly, each gene that was not within the health interval was determined as an individual dysregulation gene for a given individual. The simultaneous expression of these individual deregulated genes in each patient forms that patient's IPPGE.

### Primary Health Matrix (PHM)

The primary health matrix (PHM) is a binary matrix that serves as a tool to identify deregulated genes and assess the effectiveness of drugs in restoring their function to a healthy state. The PHM was created through the synchronization of the Institute of Public Health Post-Graduate Program (IPPGE) and Comparative Molecular Profiles of Approved Drugs (CMAP) databases [26] with the health interval. The PHM has the CMAP drugs as columns and individual deregulated genes as rows. A PHM value of 1 signifies that the gene falls within the healthy interval post-administration of the drug.

### Drug Combination Algorithm (DC algorithm)

The Drug Combination Algorithm (DC algorithm) is an algorithm that uses the PHM to determine the most appropriate drug combination for an individual patient. The algorithm has been implemented in several steps (Supplementary Table S3). Firstly, the drug that moved the most genes into their respective health intervals was selected. If several drugs equally moved the most genes into health intervals, the drug with the fewest gene expression interactions among drugs (GEIADs) was selected. GEIADs mean that a chosen drug along with previously selected drugs simultaneously affects a gene. Although the lack of GEIADs is inevitable, the algorithm can detect the fewest number of GEIADs. To identify GEIADs, we reconstructed a drug-drug network in which the drugs were defined as the nodes and GEIADs as edges. The network is detailed in Supplementary Table S3. If several drugs had the least GEIADs in equal quantity, we introduced separate drug combinations in parallel. Finally, the algorithm was completed when most genes were within health intervals with the least identified GEIADs. Pseudocodes for the DC algorithm can be found in Supplementary Table S3.

### Directed network reconstruction

To have a reference signaling pathway, all KGML Homo sapiens files were downloaded from the Kyoto Encyclopedia of Genes and Genomes (KEGG) database and merged to create a directed network using GraphPad and the Igraph package in R [27–29]. KEGG node identifiers were annotated as gene symbols.

### Gene coexpression network reconstruction

The gene coexpression networks were individually reconstructed for patients and healthy controls using the Spearman correlation. To create a differential signaling network, the directed differential coexpression network for two conditions, disease vs healthy, was detected and mapped to the reference signaling network (Fig. 2C).

### Identification of downstream targets

To identify the downstream targets of drug combinations extracted by the BMC3PM algorithm, the gene targets of the drug combinations for different patients were mapped to the differential signaling network [30]. This approach allowed us to identify the genes and pathways affected by the drug combinations and further understand the mechanism of action of these drugs.

### Visualization

All network visualizations were implemented in Cytoscape software [31], which allows for the interactive exploration and analysis of complex networks. The visualizations of the directed network and the gene coexpression network provided a comprehensive view of the molecular pathways involved in disease and the effects of drugs on these pathways.

## Results

### Individual Pattern of Perturbed Gene Expression (IPPGE)

**Fig. 3:**
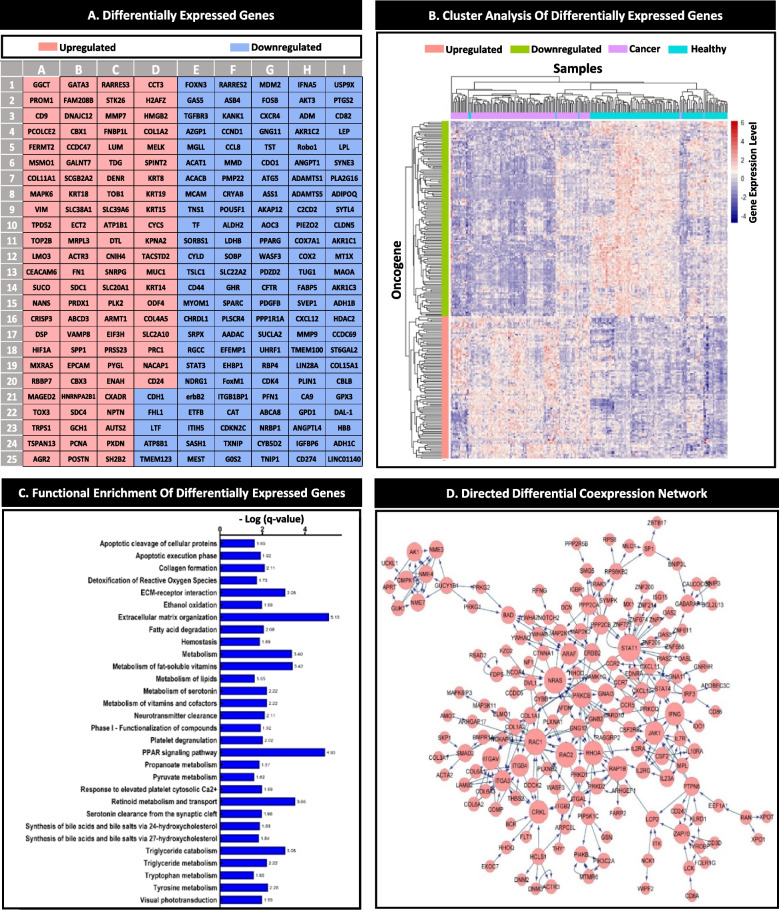
Data processing and analysis. **A** The DEGs detected from the merged data are illustrated; the total 225 genes consisted of 130 downregulated and 95 upregulated genes. These genes were used to construct the IPPGEs. **B** The heatmap shows the DEG clustering in the patient and healthy samples. The Manhattan method was used for row and column clustering. **C** The significant pathways are illustrated. The numbers on the bars indicated the minus logarithm of q-value. **D** The induced signaling pathway which was extracted from the KEGG database using the DEGs

**Fig. 4:**
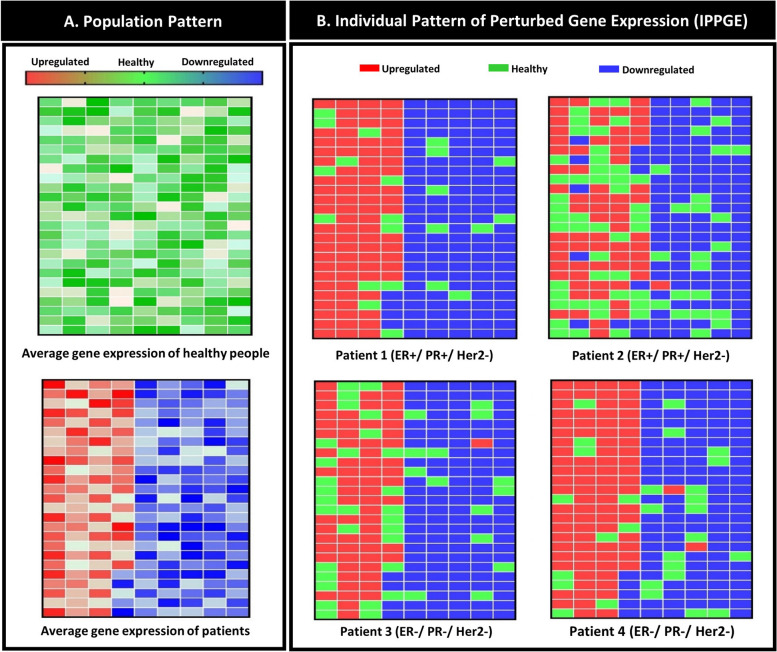
Expression pattern. **A** The expression pattern of DEGs in the population. **B** The expression pattern of DEGs in individual patients

A total of 225 genes analysis were used to construct the individual pattern of perturbed gene expression (Fig. 3A). The gene clustering and functional enrichment results were illustrated in Fig. 3B and C. A gene health interval was determined for each DEG based on comparing the gene expression between the healthy and the cancer groups. The results showed that each DEG had a unique health interval. A number of pivotal cancer-related genes, including HER2, CCND1, PIK3CA, and CDKN2A, were pinpointed within the top 500 genes in both the differential signaling network (Fig. 3D) and the analysis of differentially expressed genes. Remarkably, there were 225 genes that appeared in both analyses. As a result, certain well-known genes were left out of the study because they were not found in both analyses. The importance of genes identified as biomarkers in biological databases and previous studies is highlighted in Supplementary Table S4. Within the patient population, it was observed that the mean gene expression levels of all DEGs fell outside the health interval, as depicted in Fig. 4A. Conversely, in the healthy population, the mean gene expression of all DEGs resided within the health interval, as illustrated in Fig. 4B. In Fig. 4, each cell symbolizes one of 225 DEGs in the pattern (Fig. 3A).

Personalized studies require a unique patient profile; for this purpose, four patients in two subtypes were studied, luminal A (ER + /PR + /HER2-) and triple-negative (ER-/PR-/HER2-). The basis for constructing an IPPGE for each patient was to compare the patient’s gene expression with each DEG's health interval. Four patients were chosen at random in each subtype further to illustrate the patterns' results in this paper. The patients' gene expression patterns showed that while the first and second patients were the same in terms of subtype (ER + /PR + /HER2 +), they differed in terms of the observed IPPGE. In patient 1, 181 genes were observed to be outside of the health interval, while in patient 2, 154 genes were outside of the health interval. The third and fourth patients also had the same subtype (ER-/PR-/HER2-). In patient 3, 165 genes were outside of the health, while 169 genes were outside of the health interval in patient 4. Notably, sometimes, two patients observed an equal number of genes, but various genes were identified. Therefore, not only a significant difference was observed between the numbers of genes outside of the health interval among patients, but there was also a difference in the type of genes observed. The results indicated that patients could have very different IPPGEs despite having the same cancer subtype. Most genes in each patient were outside of the health interval, but the patients had some genes within the health interval (Fig. 4B). This study examined 6173 patients and 312 healthy people to determine their IPPGE. The results indicated that each individual had a particular IPPGE, like a fingerprint, and no two IPPGEs were identical. For some samples, the expression of 31 genes could not be assessed due to differences in the number of genes on the different platforms used in the laboratories.

### Drug combinations

The IPPGEs of the abovementioned four patients and connectivity map (CMAP) data were used as input to our designed protocol to extract personalized drug combinations. The CMAP database reports the effect of drugs on gene expression. Any drug or drug combination that could affect several IPPGE genes to bring their expression back into the health interval was extracted, forming a personalized drug combination. Given the possibility of obtaining better comparisons between breast cancer-specific drugs and between nonspecific drugs, drug combinations were extracted from these two major drug groups. The first group consisted of drugs approved for breast cancer, called the Alpha group. The second group included the Alpha group drugs and all other FDA-approved drugs and was called the Beta group. Overall, 27 combinations of drugs were extracted across the two groups for the four patients. The protocol extracted 3, 5, 3, and 2 personalized drug combinations from the Alpha group for the first, second, third, and fourth patients, respectively. From the Beta group, 3, 5, 3, and 3 personalized drug combinations were extracted for the first through fourth patients, respectively (Table 1). The third drug combination from the Alpha group for the first patient consisted of four drugs, but all of the other combinations included five drugs.

**Table 1:**
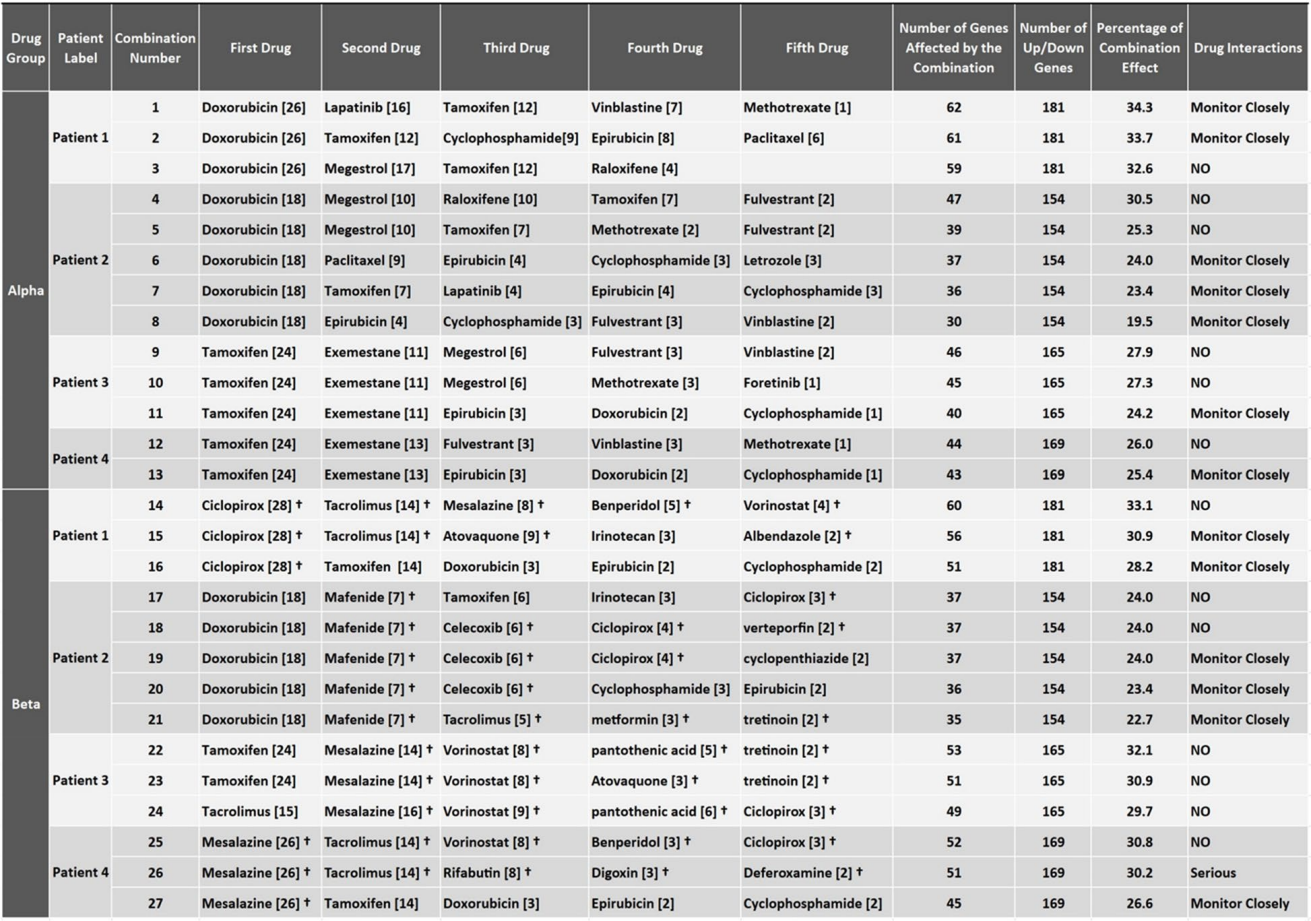
Extracted personalized drug combinations. Only specialized cancer drugs were included in the Alpha group and, thus, in all drug combinations extracted from this group, while the Beta group contained both specialized and nonspecialized drugs. Nonspecialized drugs are marked with †. The numbers within the Bracket display the number of perturbed genes for each patient that were influenced by the drug

Remarkably, each patient had a different number of genes affected by one drug. For example, gene data computations made by the algorithm indicate that in patient 1, doxorubicin affected 26 genes, while in patient 2, it affected 18 genes. In patient 2, the eight genes that doxorubicin might affect were already in the healthy interval before taking the drug. As a result, the use of this drug in patient 2 would not be efficient because it would not affect those eight genes. The difference in the effect of a drug on two separate patients was due to differences in each patient's IPPGE (Table 1).

Another intriguing observation was that after the first drug was selected, the second drug's effect under the influence of the first drug was altered. For example, tamoxifen was selected as the first drug in patient 4 in drug combination No. 13; it affected 24 genes and brought the patient's gene expression into the health interval. In comparison, in combination No. 27, tamoxifen was selected as the second drug in the same patient and was effective for 14 genes. Mesalazine, which affected 26 genes, was selected as the first drug in combination No. 27; however, tamoxifen could affect 10 of 26 genes. Consequently, genes that are jointly affected by drugs are calculated only in the drug selected in the initial step. In this way, the number of genes in a drug combination affected by each drug does not include duplicate genes (Table 1).

### IPPGEs to assess drug combinations

**Fig. 5:**
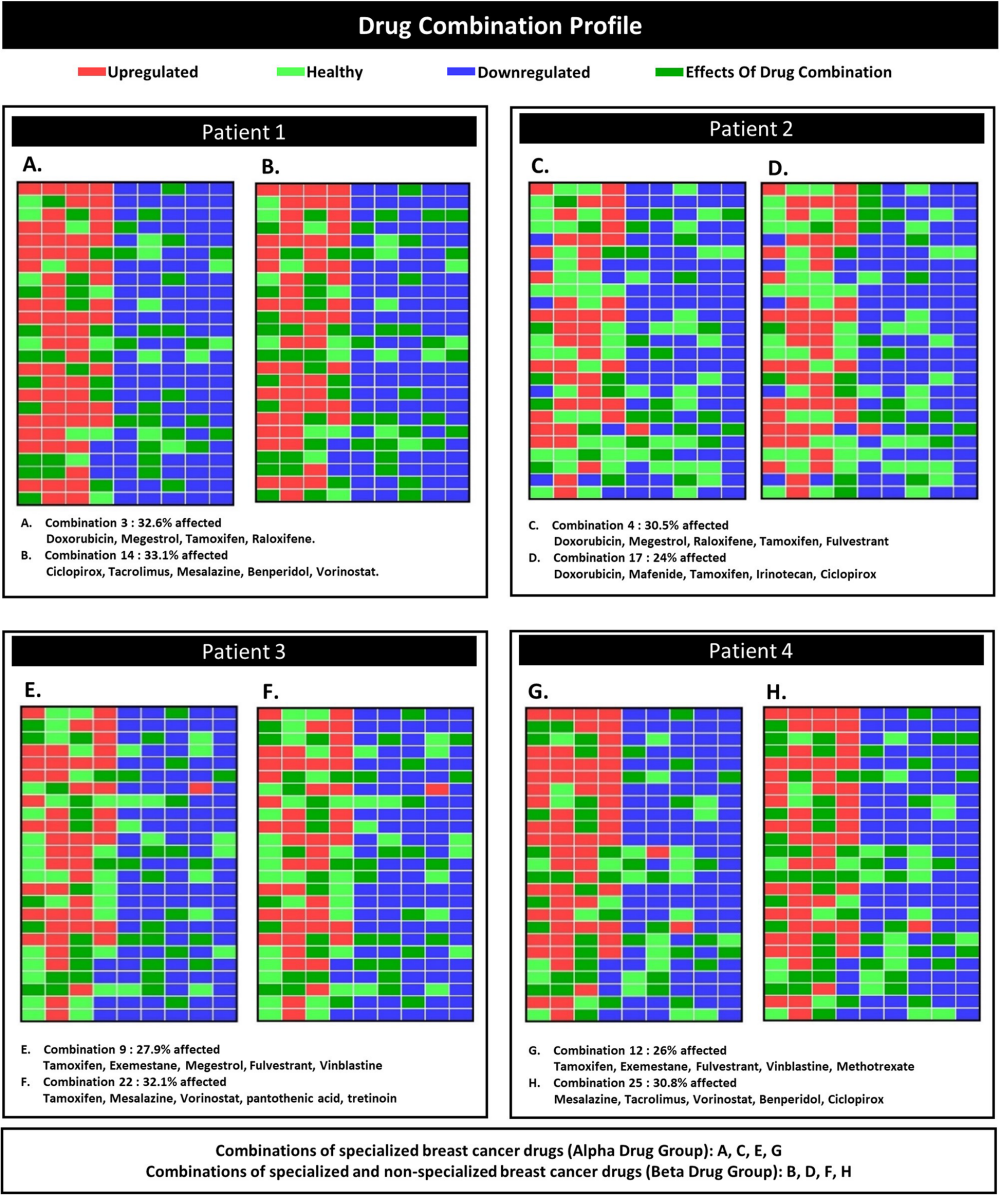
The effect of drug combinations on IPPGE. The effects of drug combinations on the gene expression of patients are illustrated. **A**, **C**, **E**, and **G** indicate the effect of specialized breast cancer-related drugs reported in Table 1 as the Alpha group. The effect of drug combinations extracted from the Beta group of drugs is shown in **B**, **D**, **F**, and **H**. The Beta group includes specialized and nonspecialized cancer drugs

The IPPGE of patients was analyzed computationally to understand the effect of the extracted drug combinations. The results showed that personalized drug combinations extracted from the Beta group were more effective than combinations extracted from the Alpha group in the second, third, and fourth patients even though all Alpha group drugs were approved breast cancer medications. Additionally, in patient 1, the most effective drug combination was from the Alpha group; however, the results showed that the combinations extracted from the Beta group had a similar effect as those from the Alpha group. The Drug Combinations Profile illustrates the effects of drug combinations on patient gene expression (Fig. 5A, B, C, D, E, F, G, and H). Moreover, Basing our analysis on DrugBank information, we classified drug interactions' side effects into three categories: serious, closely monitored, and no side effects. The outcomes demonstrated that the Beta group exhibited considerably fewer instances of side effects and drug interactions when compared to the Alpha group.

### Biological pathways to assess drug combinations

**Fig. 6:**
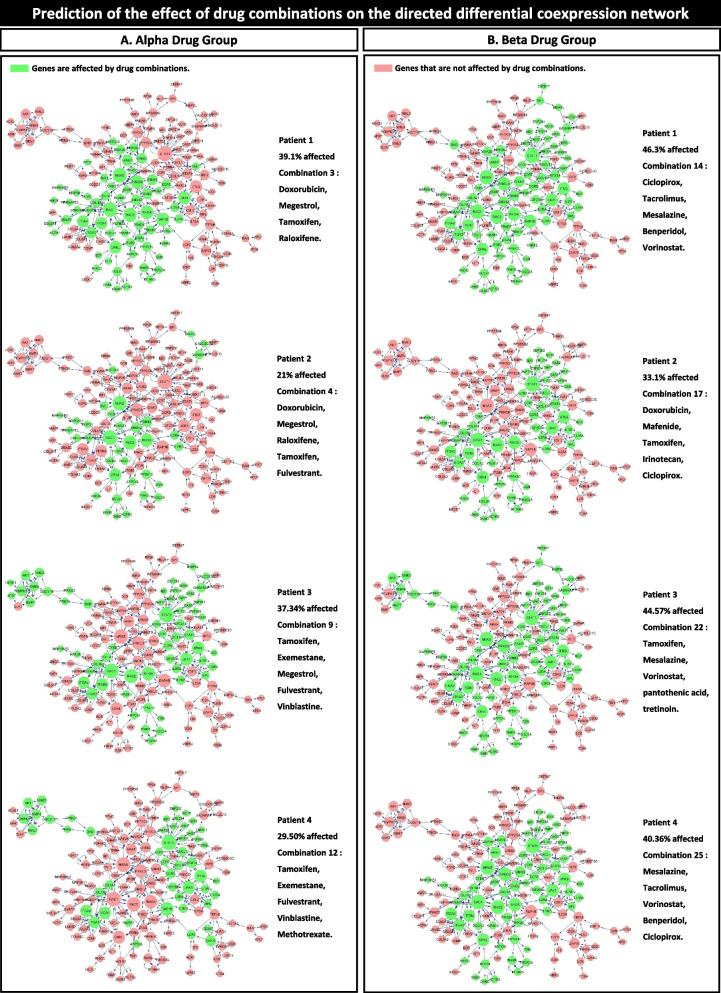
The effect of drug combinations on the directed differential coexpression network. This figure indicates the effect of the drug combinations on the signaling network. **A** Illustration of the effects of drug combinations from the Alpha group, as reported in Table 1. The Alpha group contains specialized breast cancer-related drugs. **B** The effects of the Beta group drug combinations, as reported in Table 1. Specialized and nonspecialized cancer drugs are included in the Beta group

To investigate the effect of the extracted drug combinations on biological pathways, a differential network was reconstructed from the coexpression network, which included DEGs from both healthy controls and patients (Fig. 2C). The differential network was reconstructed from an absolute weight value greater than 6.5 with 269 genes as the network node and 422 edges as the gene relationship. Using KEGG pathway data, 166 nodes and 317 edges of the differential network were able to reconstruct the directed network (Fig. 3D). For example, directional network studies for drug combinations for the four patients showed that the Beta group drug combinations were more effective than the Alpha group combinations. More efficient combinations were extracted from the Beta group, as it contains approved breast cancer-specific drugs and nonspecialized drugs. Drug combination No. 22 extracted from the Beta group for patient number 3 included tamoxifen, which is used in hormone therapy for breast cancer, and it is also included in the Alpha group. Combination No. 22 affected 44.57% of the breast cancer directional network. In contrast, drug combination No. 9 extracted from the Alpha group for the same patient could affect 37.34% of the network (Fig. 6A and B). We observed that different drugs from the same class of drugs can affect different genes in the same pathway.

The results showed that the unique IPPGEs of the patients caused different effects of each drug; thus, it was necessary to use drug combinations that were extracted based on the patient’s IPPGE to achieve the best drug effect. Although some of the drug combinations contained a joint drug, these drugs had different effects when used in different combinations. For example, ciclopirox was included in drug combinations extracted from the Beta group for patients 1, 2, and 4. Additionally, in the drug combinations extracted from the Alpha group, tamoxifen was included for all four patients. In the Beta group combinations, tamoxifen was included for patients 2 and 3 (Figs. 5, 6, and Table 1).

## Discussion

Before a new drug treatment can be employed in clinical settings, it undergoes rigorous testing and evaluation multiple times to secure approval from health organizations. However, when it comes to personalized medicine tailored for a single patient, its impact is profound for that individual alone. Consequently, the standard approval processes, which rely on tests conducted on different organisms or other individuals, are not suitable for therapies designed exclusively for one person. A recent study by Pauli and colleagues introduced a platform to address this challenge. For drug treatment validation and safety testing, this platform first applies patient‐derived tumour organoid (PDTO) cultures followed by patient-derived xenograft (PDX) models [32–35]. However, analyzing the hundreds of drugs and thousands of possible drug combinations using this platform remains a considerable and complex challenge [36]. Our new bioinformatics protocol, called BMC3PM, enabled us to extract personalized drug options that could potentially be introduced as the primary input for personalized drug treatment validation platforms.

### Designing IPPGE with a personalized medicine approach

In the present study, we created an IPPGE for each patient by comparing the gene expression interval of healthy individuals and breast cancer patients. The resulting IPPGE was unique to each patient, like a fingerprint (Fig. 4A and B). A previous study reported variations in gene expression patterns in different cancer phenotypes and also in patients [37–39]. Importantly, although patients have many common perturbed genes, gene expression compared to the health interval plays a pivotal role in drug selection [17]. Thus, the drugs and their gene targets in this study were selected in such a way as to target the most perturbed genes and not to have a useless effect on genes that were within the health interval. However, the personalized drug combinations were observed to have drugs in common among the patients to control common perturbed genes (Table 1). Therefore, each patient's unique IPPGE appeared to be effective in extracting accurate personalized drug combinations.

### Personalized medicine approach to drug combinations

Cancers are complex diseases regulated by the interaction of multiple signaling pathways through crosstalk. A single drug is thought to be capable of targeting only one signaling pathway for a disease; however, an alternative signaling pathway can be activated to maintain tumor development. A combination of drugs has been recommended to prevent drug resistance and to make the treatment more effective [40–43]. The current study also found that each medication affected a certain number of a patient's DEG genes; the algorithm extracted complementary drugs that affected the largest number of DEGgenes. We found that drug combinations derived from patient IPPGEs had stronger treatment potential due to their more targeted effects on DEG genes. In addition to the drug combination extracted for each patient, the effect of each drug alone was also recorded (Table 1). The use of drug combinations with the personalized medicine approach can lead to the identification of drug combinations that have the potential to produce a more significant effect in the patient.

### Personalized medicine approach to drug repurposing

Several drug repurposing studies have reported significant anticancer efficacy for nonspecialized drugs. One of the first drug repurposing studies showed that the anti-ulcer drug cimetidine to be a therapeutic candidate for the treatment of adenocarcinoma of the lung [44–46]. Subsequent studies have found that combination drug therapy increases the success of drug repurposing [15, 25, 47, 48]. One goal of the current study was to create a personalized medicine approach for drug repurposing. We found that drug combinations extracted from the Beta group had an equal or greater potential than those from the Alpha group for patient treatment (Figs. 5, 6, and Table 1). This finding has two distinct interpretations.

First, nonspecialized drugs may be used as adjunctive treatments in addition to specialized medications. Tamoxifen, which is used for hormone therapy in breast cancer, was the principal drug in combination No. 17. Mesalazine, vorinostat, pantothenic acid, and tretinoin were also included in this combination, and these drugs had the highest therapeutic potential as adjuvant therapy to tamoxifen for patient 3. Although these four drugs are not cancer-specific, previous reports have confirmed their anticancer effects [49–52]. Drug combinations 18 to 24 were similar to this type. These findings indicate that the therapeutic effect of the extracted personalized drug combinations can contain specialized cancer drugs in combination with nonspecialized drugs.

Second, according to the observations, some nonspecialized cancer drugs were identified as the potential main treatment. For example, ciclopirox was the first drug extracted in combinations 14, 15, and 16 for the first patient. However, this drug is a synthetic antifungal, and it is necessary to explain why none of the specific cancer drugs were found in combination No. 14. By contrast, combinations 15 and 16 contained tamoxifen and irinotecan, which are routinely used in cancer treatment [13, 53, 54]. Mesalazine was the first drug for the fourth patient in combinations 25 to 27; this drug is used to treat inflammatory bowel disease. Several other studies have reported the anticancer effects of ciclopirox and mesalazine [49, 55, 56]. Therefore, the main drug that is selected in personalized medicine may not always be a specific cancer drug—it can be extremely different based on the patient's IPPGE. The present study shows that the IPPGE can be highly unique to each patient, and the IPPGE can play an important role in extracting a personalized combination of drugs. Notably, personalized drugs with high therapeutic potential for a particular patient can include nonspecialized drugs.

In this study, we used FDA-approved drugs to assess the safety and interactions of the drug combinations, but there is great therapeutic potential among other small molecules for use as treatment options to extract future personalized drug combinations if reliable mechanisms are identified to assess drug safety and identify their interactions. This research is confined to the realm of bioinformatics, employing sophisticated in-silico methodologies for the analysis and prediction of outcomes. While our study rigorously explores computational algorithms and utilizes diverse datasets to generate insights, it is imperative to acknowledge the inherent limitations. The findings presented here have not been confirmed through laboratory experiments, thus warranting cautious interpretation. In subsequent phases of this research, comprehensive laboratory validations are indispensable to affirm the reliability and applicability of our computational predictions. These experiments are crucial steps forward, bridging the gap between theoretical analyses and practical applications, and are fundamental for the advancement of this study into tangible real-world solutions.

## Conclusion

The BMC3PM protocol was constructed in this research to extract personalized drug combinations from hundreds of drugs and thousands of drug combination. The combinations extracted included both specialized and nonspecialized cancer medications. These drug combinations can be used as the primary input for personalized medicine platforms, such as that of Pauli et al. The BMC3PM protocol can be used as a methodological interface between drug repurposing activities and combination drug therapy in a personalized precision medicine approach.

### Supplementary Information

**Additional file 1: Table S1.** Samples characteristics.

**Additional file 2: Table S2. **Drugs and datasets information.

**Additional file 3: Table S3.** Algorithms' psedocode.

**Additional file 4: Table S4.** IPPGE gene characterization.

## Data Availability

A majority of the data generated or analyzed during this study are included in this published article. The datasets generated and/or analyzed during this study can be obtained from the corresponding author upon reasonable request.
