# Correction: BMC3PM: bioinformatics multidrug combination protocol for personalized precision medicine and its application in cancer treatment

**DOI:** 10.1186/s12920-023-01784-5

**Published:** 2024-01-03

**Authors:** Majid Mokhtari, Samane Khoshbakht, Mohammad Esmaeil Akbari, Sayyed Sajjad Moravveji

**Affiliations:** 1 Department of Bioinformatics, Kish International Campus, University of Tehran, Kish Island, Iran; 2 Duke Molecular Physiology Institute, Duke University School of Medicine-Cardiology, 27701 Durham, NC USA; 3 Cancer Research Center, Shahid Beheshti University of Medical Sciences, Tehran, Iran

**Correction: BMC Med Genomics 16, 328 (2023)** 10.1186/s12920-023-01745-y

Following publication of the original article [1], it was reported that there was an error in the affiliations. Author Majid Mokhtari is only affiliated with institution 1, Department of Bioinformatics, Kish International Campus, University of Tehran, Kish Island, Iran.

The original article [1] has been corrected.
