# Supplementary material for: BMC3PM: bioinformatics multidrug combination protocol for personalized precision medicine and its application in cancer treatment
Source: BMC Med Genomics. 2023 Dec 12;16:328. doi: 10.1186/s12920-023-01745-y (PMC10717810; doi:10.1186/s12920-023-01745-y)
Supplement: Supplementary file 3 — Additional file 3: Table S3. Algorithms' psedocode. [file 12920_2023_1745_MOESM3_ESM.pdf]

Supplementary Table S 3. Algorithms' pseudocode

**Algorithm 1: Drug-Drug network reconstruction**
**Input**: Primary Health Matrix (PHM) is a binary matrix the rows and columns of which are genes and drugs, respectively. If the expression of a gene lies in the health interval, its cell in the PHM matrix would be one otherwise it would be zero.<br><br>**Output**: The DDmat is an adjacency matrix which creates an undirected weighted network. The similarity of nodes, weights of edges, indicates multiple genes that were in the health interval by every two drugs, simultaneously.

```
for (i in 1 :( number of columns (PHM)-1)) {
    d1 ← PHM [,i]
    for (k in i :(number of columns(PHM)-1)){
        d2  ← PHM [,k+1]
        tem ← 0
        for (j in 1:number of rows(PHM)) {
            if (d1 [j] == 1 AND  d2 [j]==1) tem ←  tem +1
            }
        DDmat [i,k+1] ← tem
    }
}
```

**Algorithm 2: Drug combination algorithm (DC algorithm)**
**Input**: Drug-Drug network **(**DDmat) and PHM matrixes.<br><br>**Output**: Drug combinations

```
i← 1
j←1
l← 1
sum-vector [i] ← extract sum of columns of PHM matrix
max-vector [j] ← specify the maximum of sum_vector
if (there is one maximum)
  Drug_combination[k] ← PHM [,max-vector[j]]
  K ← k+1
  (Remove the drug from PHM and call the Algorithm1 to rebuild the PHM)
Else if (there are more than one maximum)
Multi_max_vector [l] ← Extract sum of columns of the DDmat.
Min_vector ← Minimum (Multi_max_vector [l])
If ( there is one minimum)
  Drug_combination[k] ← DDmat [,Min_vector]
  K← k+1
Else (there are more than one minimum)
parallelly check all minimum and add to Drug_combination
Remove the drug from PHM and DDmat and call algorithm 1 to rebuild the
PHM
If (sum-vector[i]< 2)  the algorithm will stop.
```
